# Correction: An efficient post-processing adaptive filtering technique to rectifying the flickering effects

**DOI:** 10.1371/journal.pone.0293064

**Published:** 2023-10-12

**Authors:** Anudeep Gandam, Jagroop Singh Sidhu, Sahil Verma, N. Z. Jhanjhi, Anand Nayyar, Mohamed Abouhawwash, Yunyoung Nam

There are errors in the Funding section. The correct Funding statement is: This research was supported by Korea Institute for Advancement of Technology (KIAT) grant funded by the Korea Government (MOTIE) (P0012724, The Competency Development Program for Industry Specialist) and the Soonchunhyang University Research Fund.
